# IL-27 Activates Human Trophoblasts to Express IP-10 and IL-6: Implications in the Immunopathophysiology of Preeclampsia

**DOI:** 10.1155/2014/926875

**Published:** 2014-02-10

**Authors:** Nanlin Yin, Hua Zhang, Xin Luo, Yubin Ding, Xiaoqiu Xiao, Xiru Liu, Nan Shan, Xuemei Zhang, Qinyin Deng, Baimei Zhuang, Hongbo Qi

**Affiliations:** ^1^Department of Obstetrics and Gynecology, The First Affiliated Hospital of Chongqing Medical University, No. 1 Youyi Road, Yuzhong District, Chongqing 400016, China; ^2^Laboratory of Lipid & Glucose Research, The First Affiliated Hospital of Chongqing Medical University, Chongqing 400016, China

## Abstract

*Purpose.* To investigate the effects of IL-27 on human trophoblasts and the underlying regulatory signaling mechanisms in preeclampsia. *Methods.* The expression of IL-27 and IL-27 receptor (WSX-1) was studied in the placenta or sera from patients with preeclampsia. *In vitro*, we investigated the effects of IL-27 alone or in combination with inflammatory cytokine tumor necrosis factor (TNF-**α**) on the proinflammatory activation of human trophoblast cells (HTR-8/SVneo) and the underlying intracellular signaling molecules. *Results.* The expression of IL-27 and IL-27 receptor **α** (WSX-1) was significantly elevated in the trophoblastic cells from the placenta of patients with preeclampsia compared with control specimens. *In vitro*, IL-27 could induce the expression of inflammatory factors IFN-**γ**-inducible protein 10 (CXCL10/IP-10) and IL-6 in trophoblasts, and a synergistic effect was observed in the combined treatment of IL-27 and TNF-**α** on the release of IP-10 and IL-6. Furthermore, the production of IP-10 and IL-6 stimulated by IL-27 was differentially regulated by intracellular activation of phosphatidylinositol 3-OH kinase-AKT, p38MAPK, and JAK/STAT pathways. *Conclusions.* These results provide a new insight into the IL-27-activated immunopathological effects mediated by distinct intracellular signal transduction molecules in preeclampsia.

## 1. Introduction

Preeclampsia is a complex pregnancy-specific hypertensive syndrome, and it is a leading cause of maternal and neonatal death worldwide. As a systemic inflammation is common to all pregnancies [1], it is proposed that an excessive maternal inflammatory response to pregnancy may cause preeclampsia [2]. Besides, an angiogenic imbalance also plays an important role in the pathogenesis of preeclampsia which was associated with blood pressure, renal and endothelial dysfunction, and trophoblast deportation, as well as with a shorter duration of pregnancy, fetal growth restriction, and the severity and preterm onset of the disease in preeclampsia [3].

Recently, more studies have focused on the role of trophoblast cells which could mediate inflammation through a wide range and complex mechanisms in the development of preeclampsia. Cytokines and chemokines are the most important inflammatory mediators contributing to inflammation. In PE, trophoblast cells express inflammatory cytokines including interleukins (ILs) 1*β*, 2, 4, 6, 8, 10, 12, and 18, transforming growth factor (TGF)-beta1, IFN-*γ*-inducible protein 10/IP-10, tumor necrosis factor (TNF)-*α*, interferon (IFN)-*γ*, monocyte chemotactic protein (MCP)-1, intercellular adhesion molecule (ICAM)-1, and vascular cell adhesion molecule (VCAM)-1 [4, 5]. Among them, IL-6 is a classic multifunctional proinflammatory cytokine which is produced by the activated vascular endothelial cell and placenta [6]. The increased maternal serum levels of IL-6 are associated with the severity and onset of preeclampsia [7]. IP-10 is a cytokine of the CXC chemokine family which binds CXCR3 receptor to induce chemotaxis, apoptosis, cell growth, and angiostasis [8]. Preeclampsia was found to be associated with a higher median maternal serum concentration of IP-10 than normal pregnancy [9]. IP-10 has proinflammatory and antiangiogenic properties, and this chemokine has been proposed to be a potential link between inflammation and antiangiogenesis in preeclampsia [10]. The identification of factors and the understanding of the mechanisms regulating the production of cytokines are considered fundamental in the comprehension of the genesis in the inflammatory process of PE.

IL-27 is a heterodimeric cytokine composed of p28, an IL-12p35-related subunit, and EBI3 (Epstein-Barr virus-induced gene 3), an IL-12p40-related subunit, which belongs to IL-6/IL-12 family. IL-27 receptor is composed of the IL-27Ra (WSX-1/TCCR) subunit, unique for binding of IL-27, and the gp130 subunit which is shared with the IL-6R [11, 12]. IL-27R has been found to be expressed on monocytes, dendritic cells, T and B lymphocytes, natural killer (NK) cells, mast cells, and endothelial cells, whereas IL-27 is mainly produced by antigen-presenting cells (APC) [13, 14]. It could activate a variety of cellular targets, resulting in the production of various inflammatory mediators, including TNF-*α*, IL-1*β*, IL-6, IL-18, MIP-1*α*, MIP-1*β*, and *β*-defensin-2, thereby enhancing inflammatory reactions that can occur during some human diseases [15]. However, the role of IL-27 in the pathogenesis of PE has not been elucidated. It was therefore hypothesized that there might be an aberrant production of IL-27 in patients with PE. The aim of this study was to investigate how IL-27 activates human trophoblast cells in PE and its underlying signaling pathways.

## 2. Materials and Methods

### 2.1. Subjects

Preeclampsia (*n* = 20) and normal pregnant woman (*n* = 28) were recruited for this study, and we divided them into two groups. The matched conditions included age (±3 years), parity (0, 1–3, and 4+), and gestational age (±14 days). All cases and controls had singleton pregnancies with no known fetal abnormality. Case characteristics are detailed in Table 1.

Preeclampsia diagnosis was based on ACOG guidelines. The experiment was approved by the Clinical Research Ethics Committee of The First Affiliated Hospital of Chongqing Medical University and informed consent was obtained from all participants according to the Declaration of Helsinki.

### 2.2. Biological Samples

Placentas from caesarean section by normal and preeclamptic pregnant women were obtained from The First Affiliated Hospital of Chongqing Medical University.

Freshly obtained placentas were snap frozen immediately for processing and fixed with 10% formalin for immunohistochemistry studies. Blood samples were taken from an antecubital vein into EDTA anticoagulation tubes and then centrifuged at 4°C with a relative centrifugal force of 3000 g for 10 minutes. The serum was stored at −80°C until the analysis was performed.

### 2.3. Reagents

Recombinant human IL-12, IL-23, IL-27, TNF-*α*, and IFN-*γ* were purchased from R&D Systems (MN, USA). GAPDH antibodies were from Cell Signaling Technology (MA, USA). Mouse anti-TCCR/WXS-1 and anti-gp130 mAb were purchased from R&D Systems (MN, USA). Rabbit anti-IL-27 mAb was purchased from Abcam (HKSP, HK). Mouse anti-phospho-p38 mitogen-activated protein kinase (MAPK), anti-phospho-inhibitor(I)*κ*B-*α*, anti-phospho-Akt, anti-p38MAPK, anti-I*κ*B-*α*, and anti-Akt mAb were purchased from BD Biosciences (CA, USA). Janus kinase (JAK) inhibitor AG490, I*κ*B-*α* phosphorylation inhibitor BAY1167082, phosphatidylinositol 3-OH kinase (PI3K) inhibitor LY294002, p38MAPK inhibitor SB203580, c-Jun N-terminal kinase (JNK) inhibitor SP600125, and extracellular signal-regulated kinase (ERK) inhibitor U0126 were purchased from Calbiochem Corp. (San Diego, CA, USA). In this program, the concentration of DMSO was 0.1% (vol/vol) for all data subsets.

### 2.4. Immunohistochemistry

Formalin-fixed paraffin-embedded human placental sections were deparaffinized in xylene and then rehydrated in a series of graded alcohol. The sections were rinsed twice with PBS for 10 min and then blocked with 5% (wt/vol) nonfat milk/PBS for one hour to reduce nonspecific bindings after quenching the activity of endogenous peroxidase with 3% (vol/vol) H_2_O_2_ in PBS for 30 min. Sections were incubated with anti-IL-27 mAb (Abcam115671, HK) and anti-WSX-1 mAb (RD AF1479), diluted in 5% (wt/vol) nonfat milk for 16 h at 4°C. Negative controls were performed with the same progress. Super Sensitive Link-Label IHC detection System (BioGenex, San Ramon, CA) was used after rinsing twice with PBS and the specific immunostaining was visualized with 3,3-diaminobenzidine liquid substrate system (Sigma, St. Louis, MO). All sections were counter-stained with hematoxylin for 40 seconds and mounted with UltraKit (J. T. Baker, Deventer, The Netherlands). Five fields for each placental group were chosen at random and three placentae from each group were used.

### 2.5. Cell Culture

The HTR-8/SVneo cell line was kindly provided by Doctor CH Graham of Queen's University, Kingston, ON, Canada. Cells were grown in RPMI 1640 medium supplemented with 10% fetal bovine serum and 100 U/mL penicillin (Invitrogen Paisley, Scotland, UK) at 37°C in a 5% CO_2_ atmosphere. Cells were treated with trypsin, removed from culture flasks, and then seeded at a density of 1 × 10^6^ cells/mL. After 24–48 h culture, semiconfluent monolayers were exposed to treatments.

### 2.6. PCR

RNA was extracted from cells by Trizol reagent (Invitrogen, Carlsbad, CA), which was then followed by DNAse I digestion and reverse transcription with TaqMan Reverse Transcription Reagents (Applied Biosystems Inc., Foster City, CA, USA). The sequences of PCR primers were described in Table 2. Briefly, the reaction of quantitative real-time PCR was performed in a 25 uL volume with 2 uL cDNA, 400 nm each of sense and antisense primers, and 12.5 uL Brilliant SYBR Green QPCR Master Mix (Takara Bio Inc., Tokyo, Japan) on ABI PRISM 7000 (Applied Biosystems, Foster City, CA). The reaction performed for 40 cycles, with denaturation at 95°C for 30 seconds, annealing at 53°C for 30 seconds and extension at 72°C for 10 seconds. The gene of GAPDH was amplified as an endogenous reference. The comparative threshold cycle (CT) method was applied for relative quantification in data analysis.

### 2.7. ELISA

IL-27 was measured in serum using a human IL-27 ELISA kit with precoated plates (BioLegend, San Diego, USA). According to the manufacturer's instructions, IP-10 and IL-6 levels in culture supernatant with equal cell numbers were assayed by ELISA kit with precoated plates (R&D Systems, MN, USA). For each assay both standards and samples were tested in triplicate.

### 2.8. Western Blot

Cells and tissues were washed and lysed and an equal amount of proteins to ensure equal protein loading was subjected to SDS-PAGE and then blotted onto PVDF membrane (GE Healthcare Corp., Piscataway, NJ, USA). The membrane was blocked with 5% bovine serum albumin and probed with specific primary antibody at 4°C overnight. After washing, the membrane was incubated with secondary antibody coupled to horseradish peroxidase (GE Healthcare) for 45 minutes at 37°C. Antibody-antigen complexes were then detected using an ECL chemiluminescent detection system (GE Healthcare).

### 2.9. Statistical Analysis

All data were expressed as mean ± SD from three independent experiments. Differences between groups were analyzed by Kruskal-Wallis test, Mann-Whitney *U* test, Student's *t*-test, or one-way ANOVA analysis. To test correlations between the two parameters, the nonparametric Spearman rank correlation coefficient was used. *P* < 0.05 was considered significantly different.

## 3. Results

### 3.1. Analysis of IL-27 and IL-27 Receptor Expression in Placental Tissues from PE Patients

**Figure 1:**
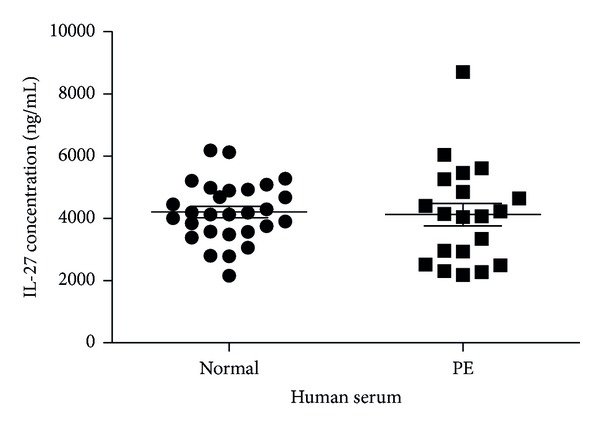
There is no significant variation in circulating IL-27 between normal and preeclamptic pregnancies. IL-27 was detected by ELISA in the serum from women with a diagnosis of preeclampsia (*n* = 20) and matched controls who had a normal pregnancy (*n* = 28). Blood samples were taken in the 3rd trimester of pregnancy. Bars represent median values.

**Figure 2:**
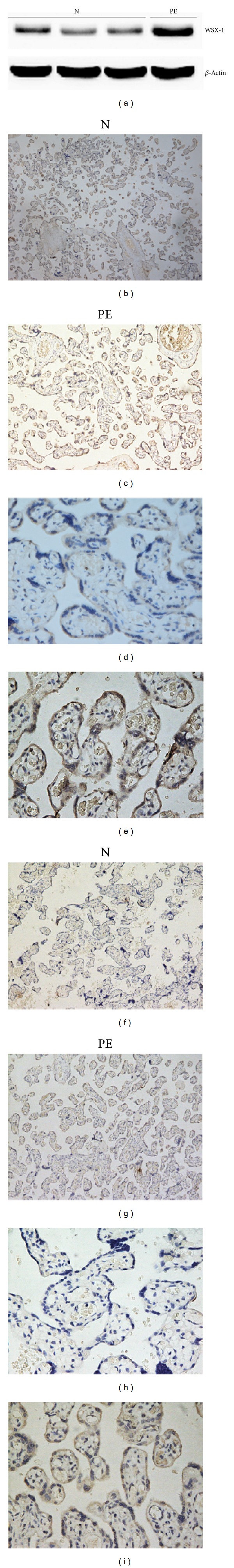
Analysis of IL-27 and IL-27 receptor expression in placental tissues. (a) Representative Western blot analysis of IL-27 receptor subunits WSX-1 in placental tissues. The first three are representative of samples of normal pregnancy (N) group and the last is from the PE group. (b)–(i) Immunohistochemical staining of IL-27 and WSX-1 in the placental tissues. (b) and (d) show the immunostaining of IL-27 in normal control (N) group; (c) and (e) show the immunostaining of IL-27 in preeclampsia (PE) group. (f) and (h) show the immunostaining of the WSX-1 in normal control (N) group; (g) and (i) show the immunostaining of WSX-1 in preeclampsia (PE) group. *β*-Actin was used as protein control to ensure an equal amount of loaded protein. Original magnification: 100x for c, f, g, and h; 400x for d, e, h, and i. All experiments were performed in three independent replicates.

IL-27 serum levels were measured in women with preeclampsia and normal pregnant women (Figure 1) and there was no significant difference between the two groups. The expression of IL-27 receptors in placental tissues of PE and control subjects was also assessed. Western blot analysis confirmed that the expression of WSX-1 in placenta tissues was significantly higher in the PE group than that in control group (Figure 2(a)). In the immunostaining of formalin-fixed paraffin-embedded serial sections, IL-27 (Figure 2(b)) and WSX-1 (Figure 2(c)) were also upregulated in the trophoblastic cells from the placenta of preeclampsia compared with control specimens.

### 3.2. IL-27 Could Upregulate IP-10 and IL-6 Expression in HTR-8/SVneo Cells

**Figure 3:**
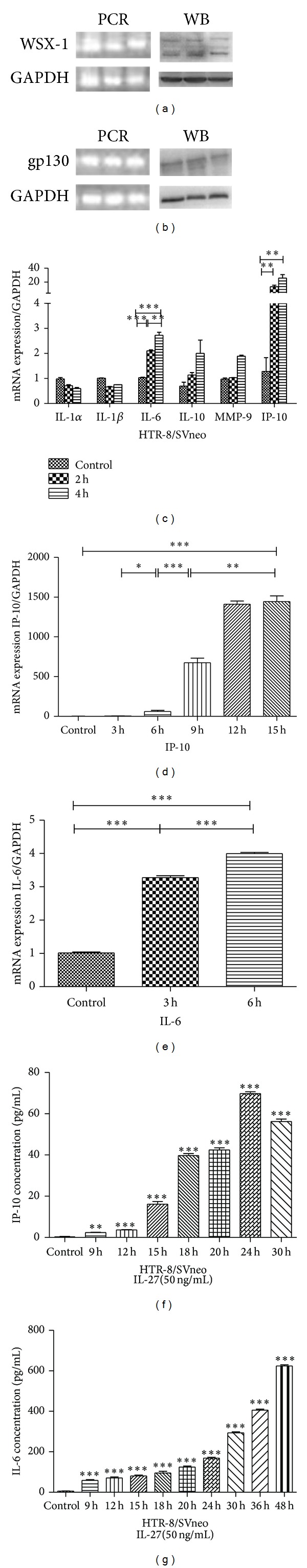
IL-27 and IL-27 receptor was expressed in HTR-8/SVneo cells and it upregulated CXCL10 and IL-6. (a) RNA and protein expression for WSX-1. (b) RNA and protein expression of gp130. From the left to the right lane, respectively, are Hct116 (human colon cancer cells), Skov3 (ovarian carcinoma cells), and HTR-8/SVneo. Hct116 and Skov3 were used as control. GAPDH was used as protein control to ensure an equal amount of loaded protein. (c) Effects of IL-27 (50 ng/mL) on the mRNA expression of inflammatory mediators. Kinetic gene expressed of CXCL10 (d) and IL-6 (e) in HTR-8/SVneo cells under stimulated with IL-27. *P < 0.05, **P < 0.01, and ***P < 0.001 when compared between groups denoted by horizontal lines (*n* = 3). (f) CXCL10 protein levels after the stimulation of IL-27 (0–50 ng/mL) in cell supernatant of HTR-8/SVneo (0–30 h). (g) IL-6 protein expression after the stimulation of IL-27 (0–50 ng/mL) in cell supernatant of HTR-8/SVneo (0–48 h). *P < 0.05, **P < 0.01, and ***P < 0.001 when comparing the level at the starting time point. All experiments were performed in three independent replicates.

Since there was an elevated expression of IL-27 and WSX-1 in the placental tissues from PE patients, we then determined the effects of IL-27 on human trophoblast cells (HTR-8/SVneo). First, it was demonstrated that the IL-27 receptor complex including WSX-1 and gp130 was constitutively expressed in human trophoblast cells on both mRNA and proteinlevels (Figures 3(a) and 3(b)). Then quantitative real-time PCR was applied to screen the inflammatory mediators expressed in trophoblast cells activated by IL-27 (50 ng/mL; 0–4 h) (Figure 3(c)). The expression of IP-10 and IL-6 was increased, while IL-1*α*, IL-1*β*, IL-10, and MMP-9 were not induced by IL-27 (Figures 3(d) and 3(e)). In addition, the protein levels of IP-10 and IL-6 in the supernatants were significantly increased (Figures 3(f) and 3(g)).

### 3.3. IL-27 and TNF-*α* Synergistically Potentiated the Production of IP-10 and IL-6 in HTR-8/SVneo

**Figure 4:**
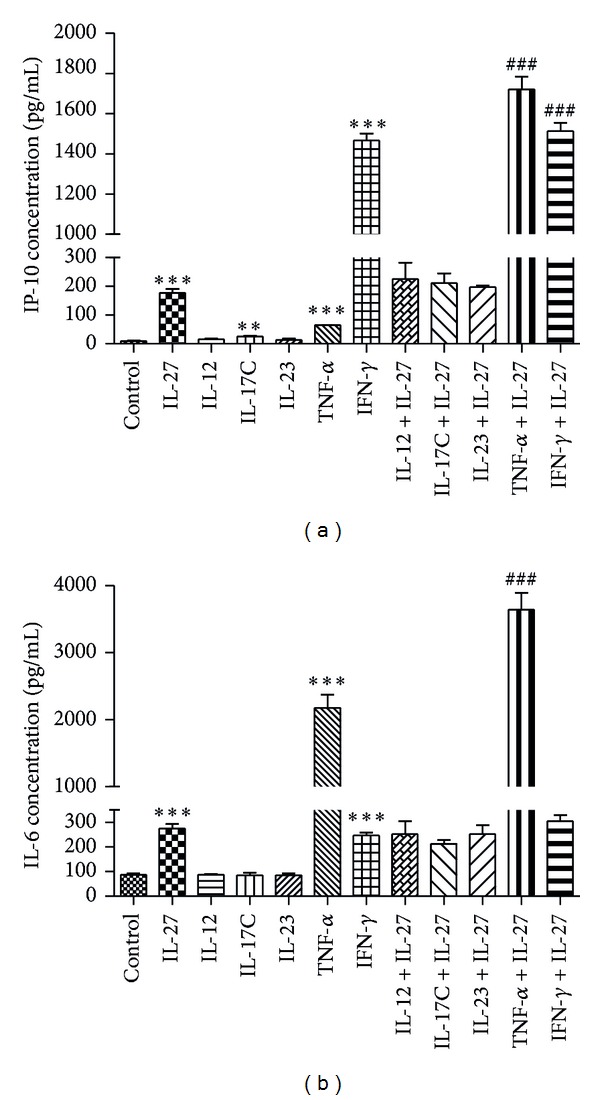
IL-27 and TNF-*α* synergistically potentiated the production of CXCL10 and IL-6 in HTR-8/SVneo. CXCL10 and IL-6 protein levels were detected by ELISA after being stimulated with cytokines. The dose for TNF-*α* and IFN-*γ* was 20 ng/mL and for other cytokines was 50 ng/mL. Results are expressed as the arithmetic mean plus SD of three independent experiments. **P < 0.01 and ***P < 0.001 when compared between treatment group and control. ^###^ *P* < 0.001 when compared between combined treatment group and IL-27 stimulated group (*n* = 3).

As many proinflammatory cytokines play a crucial inflammatory role in PE [4, 16], we investigated whether IL-27 modulates the production of IP-10 and IL-6 in HTR-8/SVneo stimulated with either a proinflammatory cytokine (TNF-*α*), IL-6/IL-12 family cytokine (IL-12 and IL-23), Th1 cytokine (IFN-*γ*) or IL-17C. The combined treatment of IL-27 and TNF-*α* resulted in a synergistic upregulation of IP-10 (176.8 ± 13.38 pg/mL for IL-27 alone; 64.75 ± 0.6059 pg/mL for TNF-*α* alone; and 1721 ± 65.02 pg/mL for IL-27 + TNF-*α*) (Figure 4(a)) and IL-6 protein expression (274.4 ± 17.76 pg/mL for IL-27 alone; 2180 ± 195.3 pg/mL for TNF-*α* alone; and 3645 ± 252.9 pg/mL for IL-27 + TNF-*α*) (Figure 4(b)). In addition, IL-27 in combination with IFN-*γ* could only additively induce IP-10 production (176.8 ± 13.38 pg/mL for IL-27 alone; 1466 ± 35.59 pg/mL for IFN-*γ* alone; 1513 ± 42.53 pg/mL for IL-27 + IFN-*γ*), and the difference of IL-6 was not significant (Figure 4(a)). However, the combination of IL-27 and IL-12, IL-23, or IL-17C did not have such a synergistic or additive effect.

### 3.4. Effects of Different Signaling Molecule Inhibitors on the Production of IP-10 and IL-6 in HTR-8/SVneo Activated by IL-27

**Figure 5:**
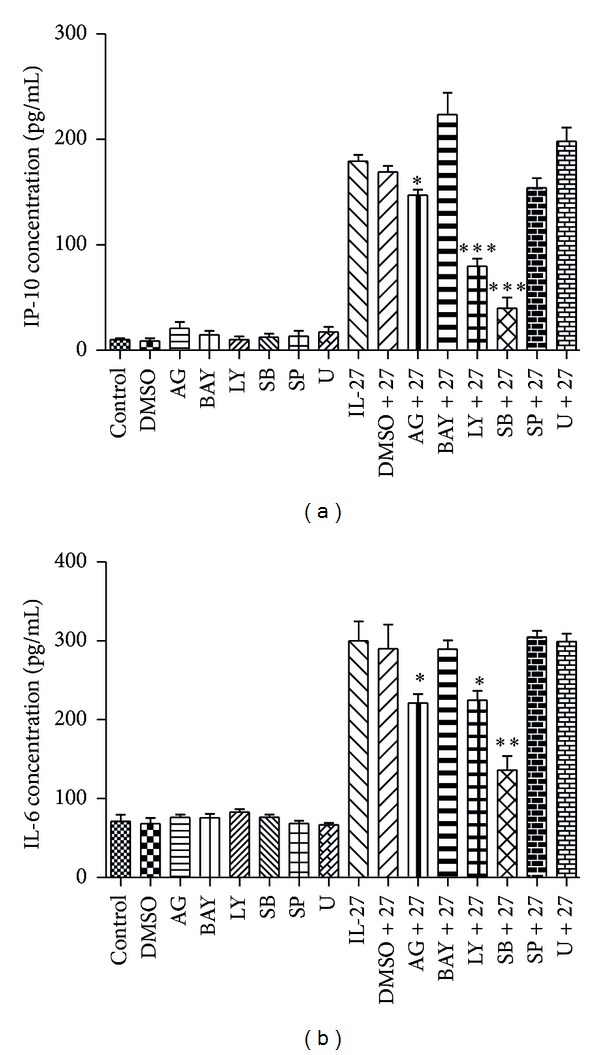
The signaling molecule inhibitors affect the production of CXCL10 and IL-6 in HTR-8/SVneo. Analysis of the effects of AG, BAY, LY, SB, SP, or U on IL-27 induced CXCL10 and IL-6 release determined by enzyme-linked immunosorbent assay. HTR-8/SVneo cells were pretreated with AG490 (9 VM; AG), BAY1167082 (0.9 VM; BAY), LY294002 (8.4 VM; LY), SB203580 (36 VM; SB), SP600125 (7 VM; SP), or U0126 (20 VM; U) for 1 h followed by incubation for a further 24 h or 48 h with or without IL-27 (50 ng/mL). DMSO (0.1%) was used as the vehicle control. Results are expressed as the arithmetic mean ± SD from three independent experiments. *P < 0.05 and ***P < 0.001 when compared between groups denoted by horizontal lines.

To study the signaling pathways which regulate the activation effects of IL-27 on HTR-8/SVneo, different signaling molecule inhibitors were applied. The toxicity threshold values of different signaling molecule inhibitors on HTR-8/SVneo were first determined by MTT assay (see Supplementary 1 in Supplementary Material available online at http://dx.doi.org/10.1155/2014/926875). The optimal concentrations were then used of JAK inhibitor AG490 (9 VM), NF-*κ*B inhibitor BAY1167082 (0.9 VM), PI3K inhibitor LY294002 (8.4 VM), p38MAPK inhibitor SB203580 (36 VM), JNK inhibitor SP600125 (7 VM), and ERK inhibitor U0126 (20 VM) with significant inhibitory effects without cell toxicity. Finally, it was found that the p38MAPK inhibitor SB203580, PI3K inhibitor LY294002, and JAK inhibitor AG490 could significantly suppress IL-27-induced IP-10 and IL-6 production in HTR-8/SVneo, whereas BAY1167082, SP600125, and U0126 did not exhibit a significant inhibitory effect (Figures 5(a) and 5(b)).

### 3.5. Effects of IL-27 on the Activation of STAT3, p38MAPK, and PI3K-Akt Signaling Pathways in HTR-8/SVneo

**Figure 6:**
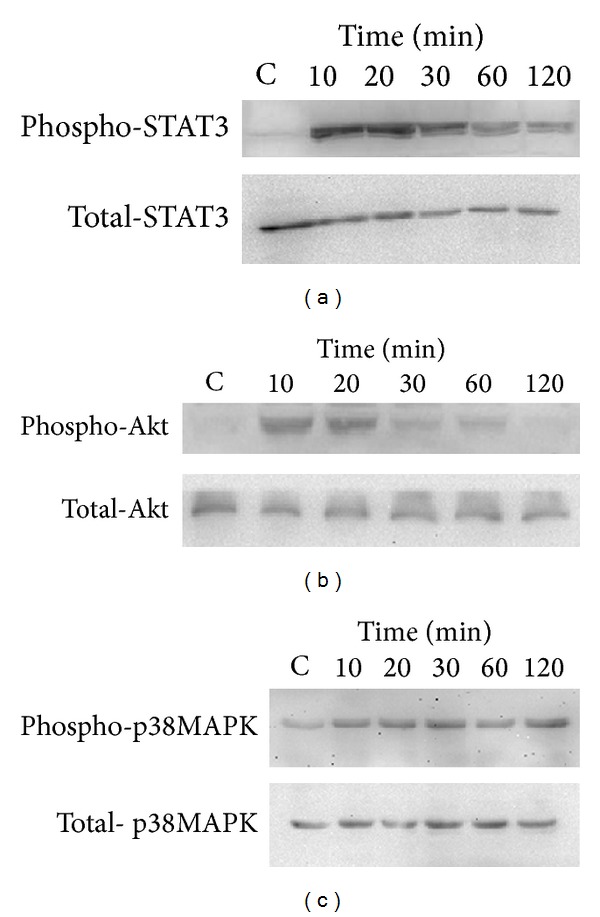
Effects of IL-27 on the activation of STAT3, p38MAPK, and PI3K-Akt signaling pathways in HTR-8/SVneo. (a) Representative Western blot analysis for the phosphorylation of STAT3. (b) Representative Western blot analysis for the phosphorylation of Akt. (c) Representative Western blot analysis for the phosphorylation of p38MAPK.

Following the inhibition assay above, it was further investigated whether IL-27 could activate JAK/STAT, p38MAPK, and PI3K-Akt signaling pathways by Western blot. IL-27 could induce significant phosphorylation of STAT3, p38MAPK, and PI3K-Akt in a time dependent manner in HTR-8/SVneo (Figures 6(a), 6(b), and 6(c)).

## 4. Discussion

In normal pregnancy, inflammation is necessary during several stages of fetal development, but it should be tightly regulated to prevent tissue injury at the fetomaternal interface. During this study, it was firstly demonstrated a dysregulated expression of IL-27 and IL-27R (WSX-1) in women with PE and that IL-27 had a proinflammatory activation on human trophoblasts, which provides a possible link between hypertensive disorders of pregnancy and inflammation.

The serum levels of IL-27 in PE patients and normal controls were determined. However, there was no significant difference between the two groups. Intriguingly, the expression of WSX-1 in placenta tissues identified by Western blot demonstrated a significant difference between PE patients and normal controls. Further immunohistochemical staining assay demonstrated that the expression of IL-27 and IL-27R*α* in the trophoblast cells from placenta tissues was significantly enhanced when compared with normal controls, suggesting that there was a dysregulated expression of IL-27 and IL-27R*α*, which may play an important role in the pathogenesis of PE.

Trophoblast cells are central participants in the pathogenesis of PE. We therefore took HTR-8/SVneo cells as a cell model to study the potential role of IL-27 in the induction of inflammatory mediators from trophoblast cells* in vitro*. It was found that IL-27 could induce a significantly higher amount of IP-10 and IL-6 in HTR-8/SVneo. In previous studies, it has been confirmed that patients with preeclampsia have significantly higher serum concentrations of IP-10 and IL-6 than normal pregnant women [7, 9]. IL-6 is involved in trophoblast invasion, proliferation, and oxidative stress, which play an important role in the pathogenesis of PE [17, 18]. IP-10 has potent antiangiogenic and promotes adhesion, migration, and invasion of trophoblast cell properties which are associated with the pathogenesis of PE [9, 19–24]. Furthermore, IL-6 and IP-10 have been shown to be highly expressed in the maternal serum of preeclampsia compared with control specimens. Here, we found that IL-27 was a novel inducer of IP-10 and IL-6 in trophoblast cells, suggesting that the IL-27- IP-10/IL-6 axis may be particularly important in the development of PE.

There are many other inflammatory mediators involved in the pathogenesis of PE [16, 25]. Therefore, we further investigated whether IL-27 could augment the production of IP-10 or IL-6 from trophoblast cells separately stimulated with proinflammatory cytokine (TNF-alpha), IL-6/IL-12 family cytokine (IL-12 and IL-23), Th1 cytokine (IFN-*γ*), or IL-17C. It was demonstrated that IL-27 could augment IP-10 and IL-6 production from trophoblast cells in combination with TNF-*α*. TNF-*α* overproduction has been observed in patients with preeclampsia [26, 27] and in animal models of preeclampsia [28], which suggests that it plays an important role in maternal physiological response observed in preeclampsia [29].

To elucidate the molecular signaling mechanisms regulating the induction of IP-10 and IL-6 from trophoblast cells by IL-27, the inhibition assay and Western bolt analysis were used to determine the signaling pathways in trophoblast cells. Using the selective specific signaling molecule inhibitors, we showed that inhibition of JAK/STAT, p38MAPK, and PI3K-Akt could partially suppress the production of IP-10 and IL-6 by IL-27, suggesting that activation of JAK/STAT, p38MAPK, and PI3K-Akt signaling pathways by IL-27 in trophoblast cells may contribute to the development of PE.

IL-27 is a proinflammatory factor which is involved in the pathogenesis of many human inflammatory diseases, such as psoriasis, arthritis, and asthma [30–32]. Therefore, IL-27 represents a potential candidate for a therapeutic approach to manage some diseases. In view of the application of antibodies for the blockade of TNF-*α*, IL-1*β*, and IL-6 receptor as treatment for RA, it was hypothesized that IL-27 may offer an alternative target for therapeutic intervention for PE [33–35]. Although many human studies are informative in describing how the immunobiology of IL-27 may be translated, it should be considered how individual polymorphisms might impact the expression patterns of IL-27. The present study provides new clues for the development of a novel treatment for IL-27-mediated PE inflammation, and further studies should investigate whether IL-27 may be a direct target for therapeutic intervention of PE.

Taken together, our results provide evidence that IL-27 could play an important role in PE. IL-27 was found to induce the expression of IP-10 and IL-6 in trophoblast cells via the activation of the JAK/STAT, p38MAPK, and PI3K-Akt signaling pathways. Elucidating the interactions between IL-27 and IP-10/IL-6 would be helpful in understanding and treating PE.

## Supplementary Material

The optimal concentrations of different specific signaling molecule inhibitors on HTR-8/SVneo were determined by MTT assay. (a)JAK inhibitor AG490 (b)NF-?B inhibitor BAY1167082 (c)PI3K inhibitor LY294002 (d) p38 MAPK inhibitor SB203580 (e) JNK inhibitor SP600125 (f)ERK inhibitor U0126. All the experiments were performed in three independent replicates.

## Figures and Tables

**Table 1:** Clinical characteristics of the third trimester study between the normal pregnant women and preeclampsia women.

Characteristic	Normal pregnant (*n* = 28)	Preeclamptic (*n* = 20)
Age (years)	26.73 ± 0.62	28.20 ± 0.73
Nulliparity %	80	72.1
BMI^a^, kg/m^2^	23.03 ± 0.46	23.45 ± 0.53
Gestation age (weeks)	37.34 ± 0.31	36.28 ± 0.27
Booking systolic BP (mmHg)	107.2 ± 1.71	124.5 ± 1.69^b^
Booking diastolic BP (mmHg)	66.21 ± 1.37	78.50 ± 1.83^c^
Max. systolic BP (mmHg)	123.7 ± 1.44	178.40 ± 15.71^b^
Max. diastolic BP (mmHg)	76.47 ± 1.13	128.30 ± 1.30^b^
24-hour proteinuria (g)	0.07 ± 0.06	2.12 ± 0.05^b^
Birth weight (g)	2962.03 ± 84.31	2512.86 ± 66.52^c^
Placental weight (g)	525.7 ± 9.36	468.3 ± 6.33^c^

Data are presented as mean ± SD unless otherwise indicated.

BMI: body mass index.

^a^BMI: body weight (kg)/body height (m^2^).

^b^ *P* < 0.01.

^c^ *P* < 0.05.

**Table 2:** Oligonucleotides used for PCR and real-time PCR.

Primer	Sequence
WSX-1:	5′-TGGACTTTTCCGAGGATGAC-3′ 3′-CTTAATGGACGACGACGAGG-5′
gp130:	5′-TGCTGATTGCAAAGCAAAAC-3′ 3′-CCTCACTTCTTCGTTCACCC-5′
IL-1*α*:	5′-AGA AGAGACGGTTGAGTTTAAGCCAATCCA-3′ 3′-ATCCAG TCGTGGAAAATCGAAGGACTC-5′
IL-1*β*:	5′-CAGGGACAGGATATGGAGCAACAA-3′ 3′-CATCTTTCAACACGCAGGACAGGT-5′
TNF-*α*:	5′-AGGCCAAGCCCTGGTATG AGC-3′ 3′-CACAGGGCAATGATCCCAAAGTAG-5′
IL-6:	5′-CACCCCTGACCCAACCACAAAT-3′ 3′-TCCTTAAAGCTGCGCAGAATGAGA-5′
IL-10:	5′-CCGCCTCAGCCTCCCAAAGT-3′ 3′-CCCTAACCTCATTCCCCAACCAC-5′
IFN-*γ*:	5′-TAGCAACAAAAAGAAACGAGATGACT-3′ 3′-GATTTTGTCCCTTCGCTTTTTCC-5′
CXCL10:	5′-TGAATCAAACTGCGATTCTG-3′ 3′-GACTTTCGTCAATCGTTCCTTT-5′
GAPDH:	5′-CAG CGACACCCACTCCTC-3′ 3′-TGTCCCACCACCTGGAGT-5′

## Abbreviations

PE: Preeclampsia
IL: Interleukin
IL-27R: IL-27 receptor
IL-27R*α*/WSX-1: IL-27 receptor *α*-chain
IP-10/CXCL10: IFN-*γ*-inducible protein 10
ICAM: Intercellular adhesion molecule
IFN: Interferon
MMPs: Matrix metalloproteinases
Th: T helper
TNF: Tumor necrosis factor
VCAM: Vascular cell adhesion molecule
TGF: Transforming growth factor
ELISA: Enzyme-linked immunosorbent assay
JAK: Janus kinase
JNK: c-Jun amino-terminal kinase
MAPK: Mitogen activated protein kinases
PI3K: Phosphatidylinositol 3-OH kinase
STAT: Signal transducers and activators of transcription
APC: Antigen-presenting cells
AG: AG490
BAY: BAY117082
LY: LY294002
SB: SB203580
SP: SP600125
U: U0126
DMSO: Dimethyl sulfoxide.
