# Supplementary material for: IL-27 Activates Human Trophoblasts to Express IP-10 and IL-6: Implications in the Immunopathophysiology of Preeclampsia
Source: Mediators Inflamm. 2014 Feb 10;2014:926875. doi: 10.1155/2014/926875 (PMC3934746; doi:10.1155/2014/926875)
Supplement: Supplementary file 1 — The optimal concentrations of different specific signaling molecule inhibitors on HTR-8/SVneo were determined by MTT assay. (a)JAK inhibitor AG490 (b)NF-?B inhibitor BAY1167082 (c)PI3K inhibitor LY294002 (d) p38 MAPK inhibitor SB203580 (e) JNK inhibitor SP600125 (f)ERK inhibitor U0126. All the experiments were performed in three independent replicates. [file 926875.f1.pdf]

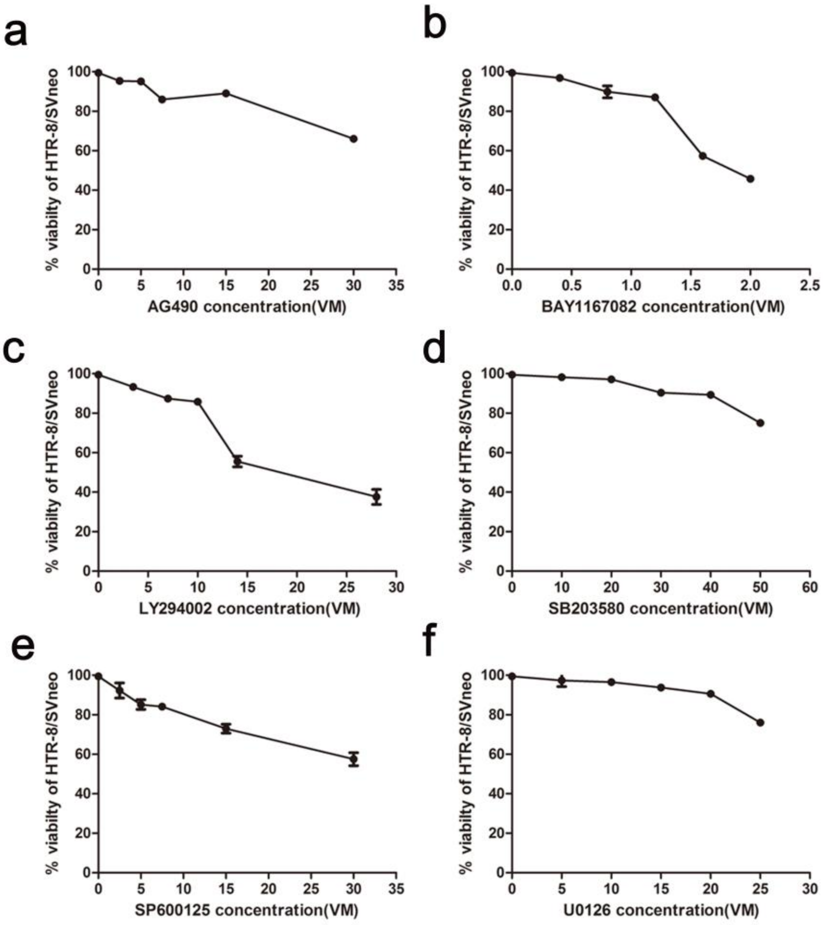

SUPPLEMENTARY1. The optimal concentrations of different specific signaling molecule inhibitors on HTR -8/SVneo were determined by MTT assay. (a)JAK inhibitor AG490 (b)NF-κB inhibitor BAY1167082 (c)PI3K inhibitor LY294002 (d) p38 MAPK inhibitor SB203580 (e)JNK inhibitor SP600125 (f)ERK inhibitor U0126. All the experiments were performed in three independent replicates.
